# Multifocal CD4+ Primary Cutaneous Small/Medium Lymphoproliferative Disorder Successfully Treated With Low-Dose Oral Methotrexate: A Case Report

**DOI:** 10.7759/cureus.8534

**Published:** 2020-06-09

**Authors:** Ahmad B Dimassi, Christopher Howlett, Chai W. Phua

**Affiliations:** 1 Internal Medicine, Gilbert and Rose-Marie Chagoury School of Medicine, Lebanese American University Medical Center, Beirut, LBN; 2 Pathology and Laboratory Medicine, Schulich School of Medicine and Dentistry, Western University, London, CAN; 3 Hematology, Schulich School of Medicine and Dentistry, Western University, London, CAN

**Keywords:** primary cutaneous cd4+ small/medium lymphoproliferative disorder, cutaneous t-cell lymphoma, multifocal, oral methotrexate

## Abstract

Primary cutaneous CD4+ small/medium T-cell lymphoproliferative disorder (PCSM-LPD) is a rare indolent disorder often associated with a favourable prognosis. It typically presents as a solitary skin lesion, mainly in the head, neck, or upper trunk region. Multifocal PCSM-LPD is a rare entity, with no standard treatment approaches available. In this article, we present the case of a 56-year-old male patient with multifocal biopsy-proven PCSM-LPD that was treated with methotrexate orally at 10 mg/m^2^ body surface area weekly and successfully achieved full clinical resolution by the 10th week of therapy. A review of the literature indicates the efficacy of combination chemotherapy. However, due to the indolent nature of this disorder and the undesired side effects from combination chemotherapy, our treatment method involved oral methotrexate alone, and it was successful. Oral methotrexate is a potential therapeutic option in the management of multifocal PCSM-LPD and it warrants further investigations.

## Introduction

Previously termed as primary cutaneous CD4+ small/medium T-cell lymphoma in the 2005 World Health Organization-European Organization for Research and Treatment of Cancer (WHO-EORTC) classification and considered a provisional entity, the condition has been recently renamed as primary cutaneous CD4+ small/medium T-cell lymphoproliferative disorder (PCSM-LPD) in the 2018 update of the WHO-EORTC classification for primary cutaneous lymphomas in light of its indolent clinical behaviour and uncertain malignant potential [1,2]. With a relative frequency of 6% among primary cutaneous lymphomas, there is no clear and established treatment modality for this disorder [1].

PCSM-LPD typically presents as a solitary plaque or tumour mainly on the face, neck, or upper trunk [3]. As per previous reports, solitary lesions were treated with local excision, intralesional steroids, radiation therapy, and occlusive fluocinolone in patches [4,5]. However, in multifocal PCSM-LPD, treatment approaches similar to the ones adopted for solitary lesions are impractical, and hence, systemic therapies such as cyclophosphamide, doxorubicin, vincristine, prednisone (CHOP) chemotherapy have been utilized [5]. In this report, we discuss a case of multifocal PCSM-LPD that was treated with oral methotrexate alone and attained a complete clinical resolution of active lesions. The decision to opt for oral methotrexate was prompted by concerns regarding the undesirable toxicities of systemic chemotherapy and the generally favorable prognosis of PCSM-LPD.

## Case presentation

A 56-year-old male patient presented with an eight-month history of multifocal subcutaneous nodules. The first nodule had appeared on his right lateral leg. Due to the persistence of the lesion, including the development of new lesions on his left arm and bilateral legs, a biopsy was pursued. He was largely asymptomatic with no constitutional B symptoms. There were no overt provoking or relieving factors. He was a lifelong nonsmoker and denied any pertinent family history. His past medical history included depression, anxiety, dyslipidemia, hypertension, gastroesophageal reflux disease, and resection of scalp basal cell carcinoma. On exam, the subcutaneous nodules were firm, immobile, non-pruritic, non-tender with a clear contour, and with the absence of overlying plaques or skin changes. The size of each lesion ranged from 2 to 3 cm. The physical exam did not reveal any palpable cervical, axillary, or inguinal lymphadenopathy, and no hepatosplenomegaly was appreciated.

**Figure 1:**
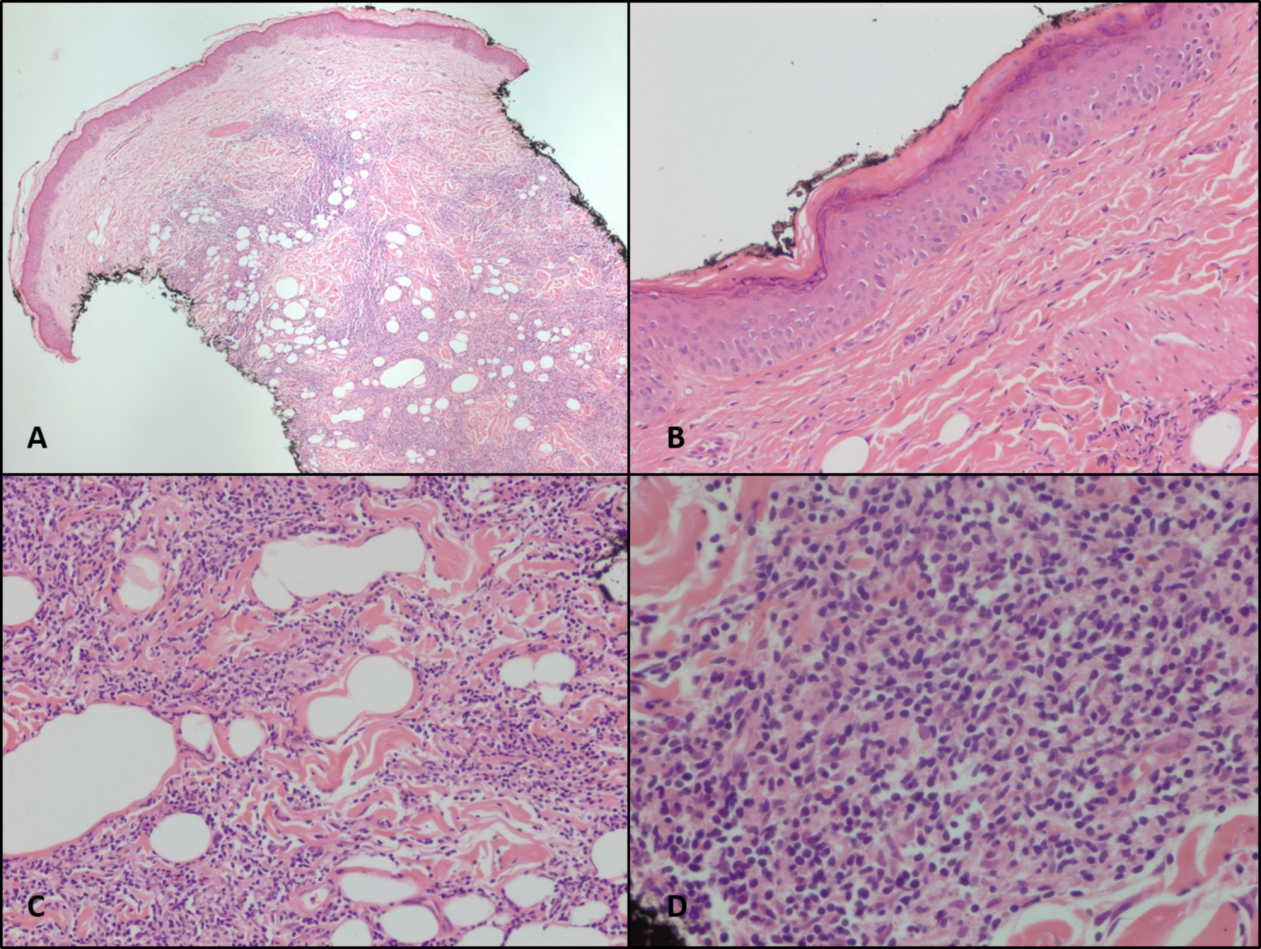
Biopsy findings - hematoxylin and eosin-stained slides (A) patchy dermal lymphocytic infiltrate at 40X magnification; (B) lack of epidermotropism at 200X magnification; (C) dermal infiltrate at 200X magnification, and (D) 400X magnification

**Figure 2:**
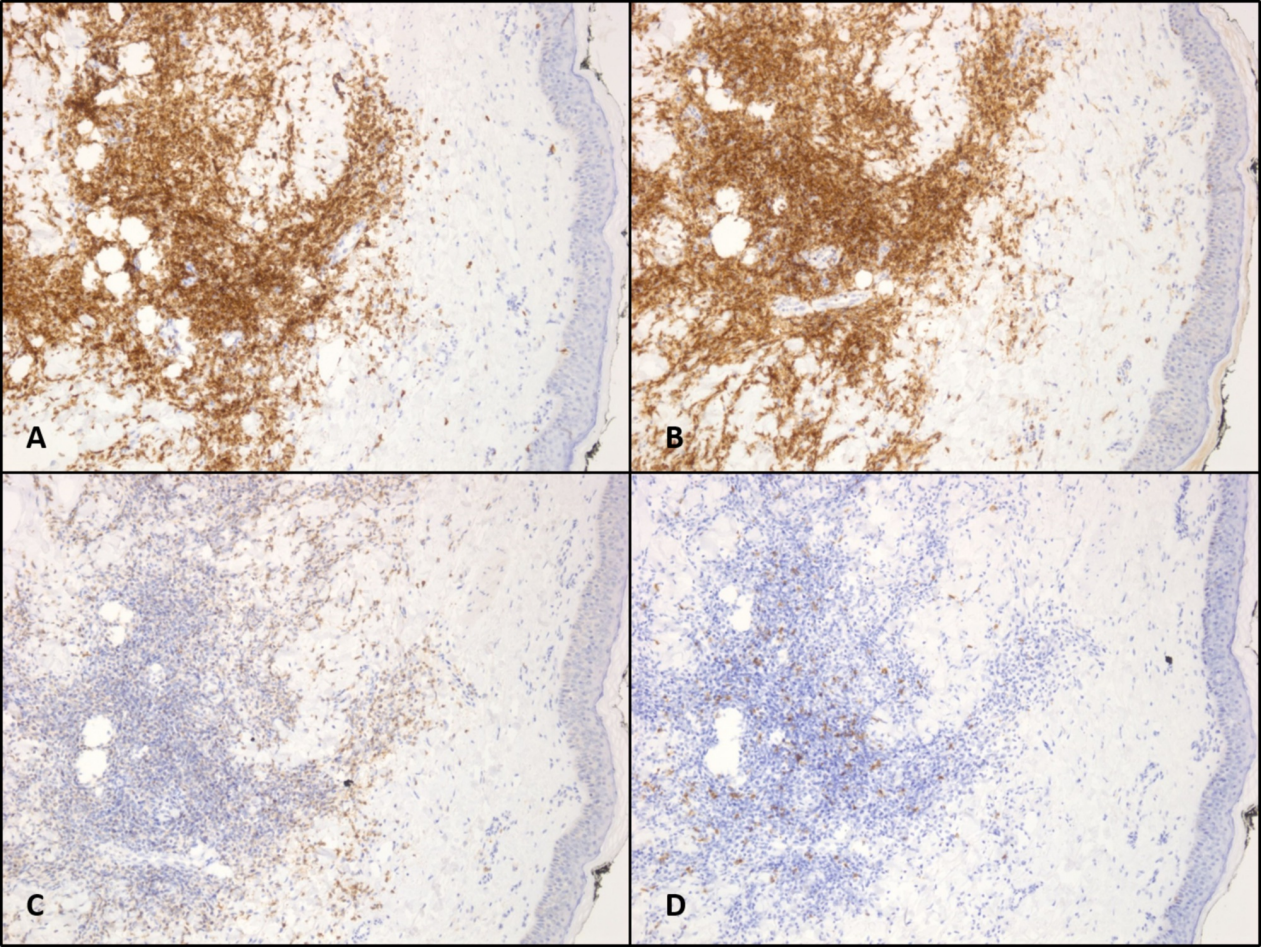
Immunophenotype of atypical lymphocytes at 200X magnification The images show positivity for CD3 (A) and CD4 (B), with partial loss of CD7 (C) and lack of CD8 expression (D)

Biopsies of both the left anterior thigh and right lateral lower leg nodules demonstrated dermal and subcutaneous infiltrate composed mostly of small lymphocytes and histocytes, with a complete absence of epidermotropism (Figure 1). Immunohistochemical studies revealed that infiltrated cells were positive for CD3, CD4, CD5, CD43, and BCL6 with weak staining for BCL2. They were negative for CD8, CD20, CD30, CD56, and PAX5. There was a loss of CD7 expression in one of the biopsies (Figure 2). In-situ hybridization for Epstein-Barr virus (EBV)-encoded RNA was negative, and the Ki67 proliferation index was estimated to be 10-20%. Special stains for acid-fast bacilli and fungal organisms were also negative.

Laboratory investigations revealed unremarkable complete blood count with differential, lactate dehydrogenase, liver enzymes, renal function, serum electrolytes, serum calcium, serum protein electrophoresis, hepatitis B and C serology, HIV antibody screen, and EBV polymerase chain reaction (PCR). Bone marrow aspirate and biopsy showed normal cellularity with no evidence of abnormal T-cell infiltrates.

**Figure 3:**
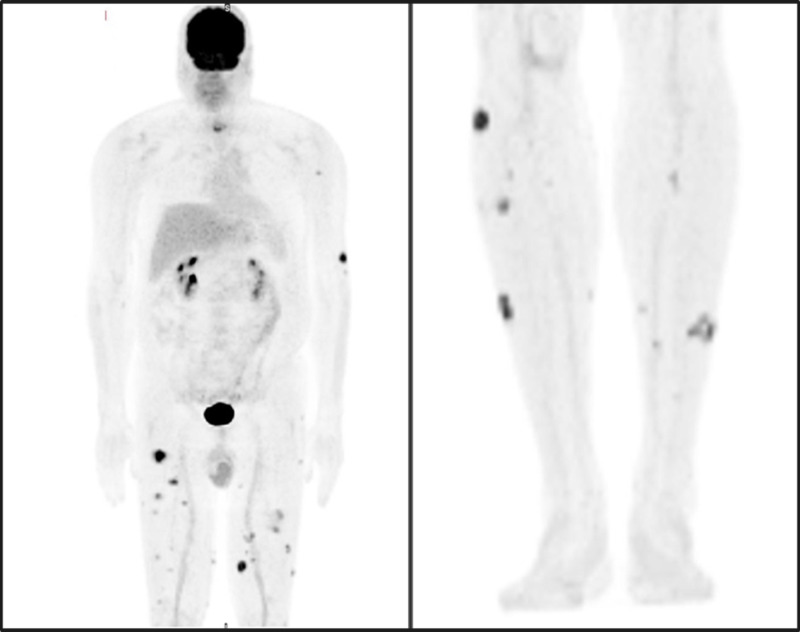
Whole-body FDG fusion PET-CT at the time of diagnosis The images show multiple FDG-avid cutaneous/subcutaneous nodules involving the left upper extremity, bilateral lower limbs, lower anterior abdominal wall, and lower back denoted by darkened areas. Uptake seen in the brain, urinary bladder, and kidneys reflect physiologic uptake FDG: F-18 fluorodeoxyglucose; PET-CT: positron emission tomography-computed tomography

A whole-body F-18 fluorodeoxyglucose (FDG) fusion positron emission tomography-computed tomography (PET-CT) done at the time of diagnosis revealed widespread hypermetabolic cutaneous and subcutaneous nodules predominantly involving the lower limbs (Figure 3). The most prominent lesion involved the patient's right anterior thigh, which measured 3.0 x 2.6 cm with a standardized uptake value (SUV) max of 13.8. The SUV uptake for abnormal cutaneous lesions ranged from 1.3 to 13.8. Aside from the cutaneous lesions as seen on PET-CT, there was no additional systemic involvement seen on imaging.

The patient was diagnosed with multifocal PCSM-LPD and was initiated on oral methotrexate 25 mg once a week (dosed at 10 mg/m^2^ body surface area weekly). Response to therapy was evident within four weeks with partial flattening of cutaneous lesions. By the 10th week of weekly methotrexate initiation, the patient had full resolution of his skin nodules with the absence of new lesions. He remained in complete clinical response at his last follow up visit, which was about 12 months from the start of oral methotrexate. To date, he remains on weekly oral methotrexate with no significant adverse events.

## Discussion

PCSM-LPD remains a provisional entity in the recent 2018 WHO-EORTC classifications for primary cutaneous lymphomas due to its uncertain malignant potential. Unlike our case presentation, it classically presents as a solitary nodule of the face, neck, or trunk [6]. In a recent systematic review, solitary lesions as the initial presentation were found in about 80% of the cases, mostly involving the head and neck. Multiple lesions were reported in only 8.31% of the cases. The median age of diagnosis was 53 years [7]. In general, the prognosis of PCSM-LPD is deemed favourable, especially if presenting with a solitary lesion, although some studies suggest worse outcomes if lesions are multifocal, larger than 5 cm, and have a decreased expression of CD4 and higher proliferation index [2,8]. It is unclear if these cases represented peripheral T-cell lymphoma, unspecified, instead of PCSM-LPD.

On pathologic evaluation, PCSM-LPD reveals dense lymphocytic infiltration of the dermis and subcutis with small-medium sized lymphocytes with mild pleomorphism. They typically express CD3, CD4, CD5, PD1, and CXCL 13 with variable BCL6. They lack expression of cytotoxic proteins, CD10 and CD30. Other than CD7, the loss of pan T-cell markers is unusual [9-10]. A minority of large pleomorphic T cells may also be present but often make up less than 30% of all cells, which may help to distinguish from peripheral T-cell lymphoma not otherwise specified [11]. Unlike mycosis fungoides, which share many histological similarities with PCSM-LPD, there is an absence of epidermotropism. Based on our patient’s clinical presentation and pathologic findings, his diagnosis was most consistent with multifocal PCSM-LPD.

Local therapy for solitary nodules, such as surgical excision, local radiation, or intralesional steroids, often leads to excellent response [12]. There are, however, no clear treatment approaches for multifocal PCSM-LPD. The idea of performing local therapy in a patient with multifocal PCSM-LPD is impractical. A case report published by Choi et al. features a patient diagnosed with PCSM-LPD and presenting with multifocal nodules on the face, abdomen, and leg. Local radiation therapy was initially attempted. However, there was an early relapse at a non-radiated site about one month from therapy completion. Therefore, the patient was started on systemic CHOP chemotherapy with complete remission of the skin lesions after the first cycle [13].

As for our case, since our patient was largely asymptomatic with no physical symptoms aside from the chronic persistent skin nodules, we decided to utilize low-dose oral methotrexate, which had shown efficacy in other indolent T-cell disorders, instead of systemic chemotherapy that could potentially lead to unfavorable toxicities. Methotrexate dosed orally at 10 mg/m^2^ weekly, with daily folic acid supplements except on the day of methotrexate, led to a clinical response by week four and complete resolution of all his skin nodules by week 10.

## Conclusions

We discussed a case of multifocal PCSM-LPD in a 56-year-old male patient that was successfully treated with low-dose oral methotrexate. A complete clinical resolution of his skin lesions was achieved. Low-dose oral methotrexate was well tolerated with minimal toxicities. The patient remained in complete clinical response with no recurrence of disease at his last follow up visit about 12 months post-initiation of oral methotrexate. Moving forward, we intend to provide a trial of methotrexate discontinuation with close clinical monitoring for a potential relapse. Oral methotrexate as a therapeutic modality in multifocal PCSM-LPD warrants further investigations.
